# 737. Geographic Clustering of Travel-acquired Infections in Ontario, Canada, 2008-2020

**DOI:** 10.1093/ofid/ofab466.934

**Published:** 2021-12-04

**Authors:** Vinyas Harish, Emmalin Buajitti, Holly Burrows, Joshua Posen, Isaac Bogoch, Jonathan Gubbay, Andrea Boggild, Andrea Boggild, Laura Rosella, Shaun Morris

**Affiliations:** 1 University of Toronto, Toronto, Ontario, Canada; 2 Yale University, New Haven, Connecticut; 3 Hospital for Sick Children, Toronto, Ontario, Canada; 4 Hospital for Sick Children, University of Toronto, Toronto, Ontario, Canada

## Abstract

**Background:**

As rates of international travel increase, more individuals are at risk of travel-acquired infections (TAIs). We aimed to review all microbiologically confirmed cases of malaria, dengue, chikungunya, and enteric fever (*Salmonella enterica* serovar Typhi/Paratyphi) in Ontario, Canada between 2008-2020 to identify high-resolution geographical clusters that could be targeted for pre-travel prevention.

**Methods:**

Retrospective cohort study of over 174,000 unique tests for the four above TAIs from Public Health Ontario Laboratories. Test-level data were processed to calculate annual case counts and crude population-standardized incidence ratios (SIRs) at the forward sortation area (FSA) level. Moran’s I statistic was used to test for global spatial autocorrelation. Smoothed SIRs and 95% posterior credible intervals (CIs) were estimated using a spatial Bayesian hierarchical model, which accounts for statistical instability and uncertainty in small-area incidence. Posterior CIs were used to identify high- and low-risk areas, which were described using sociodemographic data from the 2016 Census. Finally, a second model was used to estimate the association between drivetime to the nearest travel clinic and risk of TAI within high-risk areas.

**Results:**

There were 5962 cases of the four TAIs across Ontario over the study period. Smoothed FSA-level SIRs are shown in Figure 1a, with an inset for the Greater Toronto Area (GTA) in 1b. There was spatial clustering of TAIs (Moran’s I=0.61, p< 2.2e-16). Identified high- and low-risk areas are shown in panels c and d. Compared to low-risk areas, high-risk areas were significantly more likely to have higher proportions of immigrants (p< 0.0001), lower household after-tax income (p=0.04), more university education (p< 0.0001), and were less knowledgeable of English/French (p< 0.0001). In the high-risk GTA, each minute increase in drivetime to the closest travel clinic was associated with a 4% reduction in TAI risk (95% CI 2 - 6%).

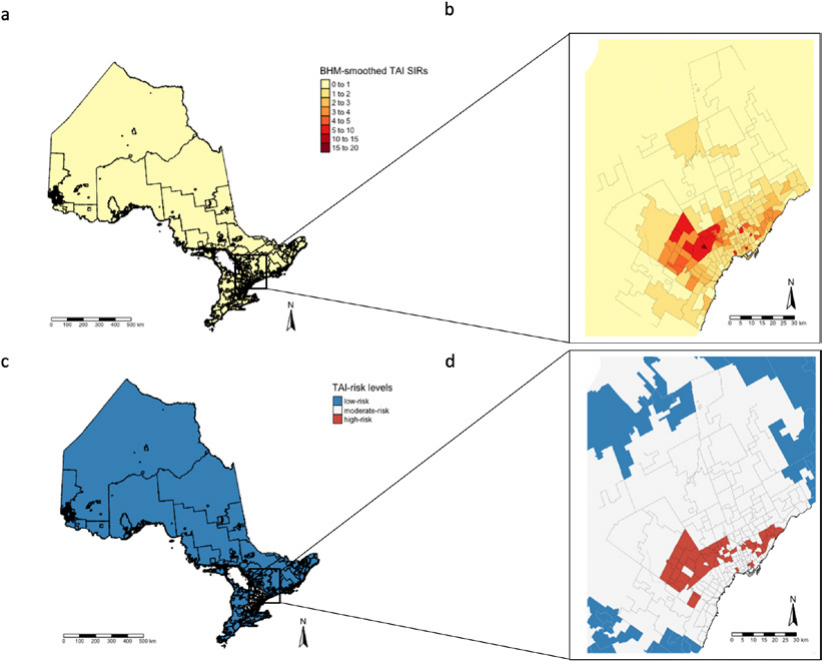

Bayesian hierarchical model (BHM) smoothed standardized incidence ratios (SIRs) for travel-acquired infections (TAIs) and estimated risk levels (a and c) with insets for the Greater Toronto Area (b and d). High-risk areas are defined as those with smoothed SIR 95% CIs greater than 2, and low-risk areas with smoothed SIR 95% CIs less than 0.25.

**Conclusion:**

Urban neighbourhoods in the GTA had elevated risks of becoming ill with TAIs. However, geographic proximity to a travel clinic was not associated with an area-level risk reduction in TAI, suggesting other barriers to seeking and adhering to pre-travel advice.

**Disclosures:**

**Isaac Bogoch, MD, MSc**, **BlueDot** (Consultant)**National Hockey League Players' Association** (Consultant) **Andrea Boggild, MSc MD DTMH FRCPC**, Nothing to disclose **Shaun Morris, MD, MPH, DTM&H, FRCPC, FAAP**, **GSK** (Speaker's Bureau)**Pfizer** (Advisor or Review Panel member)**Pfizer** (Grant/Research Support)

